# Both HCV Infection and Elevated Liver Stiffness Significantly Impacts on Several Parameters of T-Cells Homeostasis in HIV-Infected Patients

**DOI:** 10.3390/jcm9092978

**Published:** 2020-09-15

**Authors:** Clara Restrepo, Beatriz Álvarez, José L Valencia, Marcial García, María A Navarrete-Muñoz, José M Ligos, Alfonso Cabello, Laura Prieto, Sara Nistal, María Montoya, Miguel Górgolas, Norma Rallón, José M Benito

**Affiliations:** 1 HIV and Viral Hepatitis Research Laboratory, Instituto de Investigación Sanitaria Fundación Jiménez Díaz, Universidad Autónoma de Madrid (IIS-FJD, UAM), 28933 Madrid, Spain; clararestrepog@gmail.com (C.R.); marcial_g_a@hotmail.com (M.G.); mangelesnm94@gmail.com (M.A.N.-M.); jbenito1@hotmail.com (J.M.B.); 2 Hospital Universitario Rey Juan Carlos, Móstoles, 28933 Madrid, Spain; sara.nistal@hospitalreyjuancarlos.es; 3 Hospital Universitario Fundación Jiménez Díaz, 28040 Madrid, Spain; BAlvarez@quironsalud.es (B.Á.); acabello@fjd.es (A.C.); LPrietoPe@fjd.es (L.P.); mgorgolas@fjd.es (M.G.); 4 Departamento de Estadística e Investigación Operativa III, Facultad de Estudios Estadísticos, Universidad Complutense de Madrid, 28040 Madrid, Spain; joseval@estad.ucm.es; 5 Centro Nacional de Investigaciones Cardiovasculares (CNIC), 28029 Madrid, Spain; jmligos@gmail.com (J.M.L.); mmontoya@cnic.es (M.M.)

**Keywords:** HIV/HCV coinfection, T-cell homeostasis disturbances, liver stiffness, immune restoration, DAAs-based therapy

## Abstract

(1) Background: The role of hepatitis C virus (HCV) co-infection on the T-cell homeostasis disturbances in human immunodeficiency virus (HIV)-infected patients as well as its reversion after HCV eradication with direct acting antivirals (DAAs) therapy has not been yet clarified. We extensively analyzed the effect of HCV co-infection on immune parameters of HIV pathogenesis and its evolution after HCV eradication with DAAs. (2) Methods: Seventy individuals were included in the study—25 HIV-monoinfected patients, 25 HIV/HCV-coinfected patients and 20 HIV and HCV seronegative subjects. All patients were on antiretroviral therapy and undetectable HIV-viremia. Immune parameters, such as maturation, activation, apoptosis, senescence and exhaustion of T-cells were assessed by flow cytometry. Cross-sectional and longitudinal (comparing pre- and post-DAAs data in HIV/HCV coinfected patients) analyses were performed. Univariate and multivariate (general linear model and canonical discriminant analysis -CDA-) analyses were used to assess differences between groups. (3) Results—The CDA was able to clearly separate HIV/HCV coinfected from HIV-monoinfected patients, showing a more disturbed T-cells homeostasis in HIV/HCV patients, especially activation and exhaustion of T-cells. Interestingly, those perturbations were more marked in HIV/HCV patients with increased liver stiffness. Eradication of HCV with DAAs restored some but not all the T-cells homeostasis disturbances, with activation and exhaustion of effector CD8 T-cells remaining significantly increased three months after HCV eradication. (4) Conclusions—HCV co-infection significantly impacts on several immune markers of HIV pathogenesis, especially in patients with increased liver stiffness. Eradication of HCV with DAAs ameliorates but does not completely normalize these alterations. It is of utmost relevance to explore other mechanisms underlying the immune damage observed in HIV/HCV coinfected patients with control of both HIV and HCV replication.

## 1. Introduction

The UNAIDS (Joint United Nations Programme on HIV and AIDS) estimates that around 37.9 million people were living with human immunodeficiency virus (HIV) in 2018 [1] and 71 million people were chronically infected with hepatitis C virus (HCV) [2]. Since both viruses are transmitted by contaminated blood products, around 2.3 million people are coinfected with HIV and HCV [2] and the main risk groups are people who inject drugs (PWID) followed by men who have sex with men (MSM) [3,4,5]. Co-infection poses an additional stress on host immune system and, as a consequence, each virus can aggravate the normal clinical course of the concomitant infection. It is well known the negative impact of HIV on the HCV progression—higher levels of HCV load [6], faster liver disease progression [7,8] and hepatocellular carcinoma (HCC) [9]. All these clinical conditions improve with the use of antiretroviral therapy (ART) with a reduction of HCV viral load, better liver-related survival and lower rates of fibrosis development [8,10,11]. Studies on the potential effect of HCV on clinical progression of HIV are scarcer and more controversial [12,13,14,15]. 

The effect of HCV on immune parameters of HIV infection pathogenesis has also been analyzed. Most previous studies have focused on T cell activation and apoptosis [16,17,18,19,20,21,22], some of them reporting discordant results [20,21,22]. Other aspects of HIV pathogenesis such as T cell exhaustion, senescence, maturation and thymic function have been less explored in HIV/HCV co-infection [18,22,23,24,25]. In a large cohort of HIV/HCV patients naive for both anti-HCV treatment and for ART and using a cross-sectional design, we found that HIV pathogenesis was significantly affected by HCV, specially activation and exhaustion of CD8 T-cells [26].

The impact of HCV on HIV pathogenesis has also been explored in cohorts of HIV/HCV patients undergoing anti-HCV treatment. Most previous studies have analyzed only a limited number of parameters, focusing mainly on T cell activation [17,27,28] or apoptosis [21], in cohorts of patients undergoing anti-HCV treatment with IFN-α and ribavirin and thus the results obtained maybe biased by the immunomodulatory effect of IFN-α [29]. The current therapy for HCV, based on the use of direct acting antivirals (DAAs) without any immunomodulatory effect and with very high potency to eradicate HCV [30], brings us the opportunity to better clarify the role of HCV on HIV pathogenesis. For this purpose, herein we have performed an in-depth analysis of several immune parameters related to HIV infection pathogenesis, in a well-defined cohort of HIV/HCV coinfected patients before and after HCV eradication with the new DAAs-based therapy.

## 2. Experimental Section

### 2.1. Study Population

Patients included in the study were recruited from the infectious diseases outpatient clinic of Hospital Universitario Fundación Jiménez Díaz, Madrid. Two groups were included—twenty-five HIV patients coinfected with HCV (HIV/HCV group) and 25 HIV monoinfected patients (HIV group). Twenty age and sex-matched HIV and HCV-seronegative individuals from blood banking were included as seronegative controls (SNC group), as a reference group for the T-cells subsets evaluated. Inclusion criteria for HIV group were—adults (>18 years of age) with chronic HIV infection, being on ART and having undetectable plasma HIV-RNA for at least 12 months prior to inclusion. Inclusion criteria for HIV/HCV group were the same as those for HIV group and also to have chronic active HCV infection (positive HCV serology and detectable levels of plasma HCV-RNA) and being eligible for anti-HCV treatment with IFNα/ribavirin-free DAAs-based regimens. All subjects included in the study signed a written informed consent and the study protocol was approved by the ethical committee of the Hospital Universitario Fundación Jiménez Díaz (PIC 46/2015, record number 07/15 dated on 14 April 2015).

### 2.2. Cell Samples

EDTA-anticoagulated blood was obtained by venipuncture and peripheral blood mononuclear cells (PBMCs) were immediately isolated by density gradient centrifugation using Ficoll-Hypaque (Sigma Chemical Co., St. Louis, MO, USA) and frozen in fetal calf serum (FCS) plus 10% dimethyl sulfoxide (DMSO). For HIV patients and seronegative controls a single sample was obtained at the moment of inclusion. For HIV/HCV group two different samples were obtained—at the moment of inclusion before starting anti-HCV treatment (pre-DAAs HIV/HCV group) and 3 months after the end of treatment, when sustained virological response (SVR) is evaluated (post-DAAs HIV/HCV group). Cryopreserved PBMCs were employed for immunophenotypic analysis. Viability of thawed PBMCs was always greater than 85%.

### 2.3. Immunophenotypic Analysis

Multiparameter flow cytometry was employed to analyze the expression levels of 182 unique T-cells subsets. PBMCs were stained with a panel of thirteen different monoclonal antibodies that in combination defined a total of 182 unique T cell subsets. The following parameters were analyzed in CD4 and CD8 T cells—maturation (measuring CD45RA and CCR7 expression), activation (measuring CD38 and HLADR expression), exhaustion (measuring PD1 and Tim3 expression), senescence (measuring CD28 and CD57 expression), apoptosis (measuring CD28 and CD95 expression), recent thymic emigrants (measuring CD31 expression). The complete list of monoclonal antibodies and fluorochromes used in the study is shown in Supplementary Table S1. The staining procedure and flow cytometry analysis are described in Supplementary Methods and a representative example of flow cytometry analysis is shown in Supplementary Figure S1. The complete list of all T cell subsets analyzed with their levels of expression in the different study groups are shown in Supplementary Table S2.

### 2.4. Statistical Analysis

The main characteristics of the study groups were expressed as median [interquartile range] or as proportion and the comparisons between groups were done using Mann-Whitney U-test or chi-squared test as appropriate. Inter and intra-group differences in the levels of T cell subsets were tested using a univariate analysis (Kruskall-Wallis test, Mann-Whitney U test and Wilcoxon signed-rank test as appropriate) and the effect of different covariates was adjusted using a general linear model (GLM). Moreover, a partial least square regression (PLS) was employed to reduce the dimensionality of data followed by a canonical discriminant analysis to separate between different groups of subjects (HIV patients, HIV/HCV patients and SNC individuals) and between different timepoints (pre and post-therapy in HIV/HCV patients). A detailed description of discriminant analysis is given in Supplementary Methods.

## 3. Results

### 3.1. Characteristics of Patients Included in the Study

The main characteristics of patients at the time of inclusion in the study are shown in Table 1. All HIV/HCV patients were naïve for anti-HCV therapy at the time of inclusion. There were no significant differences between HIV and HIV/HCV groups in terms of age, gender, time since HIV diagnosis, time on ART, CD4 counts and HIV transmission route. The group of seronegative controls (SNC) was matched with the groups of patients for age (46 (43–51) years, *p* = 0.175 for the global comparison between the study groups) and for gender (100% of SNC donors were male, *p* = 0.24 for the comparison between SNC and HIV groups). AST and ALT hepatic enzymes were significantly higher in HIV/HCV patients, whereas total cholesterol and LDL were significantly higher in HIV patients. Liver stiffness was <7.1 KPa in 60% of patients and >7.1 KPa in 40%. According to the study by Cástera et al. [31], this cut-off of 7.1 KPa separates between F0–F1 (<7.1 KPa) stages of the Metavir fibrosis scale (no significant fibrosis) and F2–F4 (>7.1 KPa) stages of Metavir scale (significant fibrosis). Regarding the DAAs combinations used, because all HIV/HCV coinfected patients were recruited between 2015–2018 and according to the DAAs combinations available on those dates for the treatment of HCV genotypes 1 and 4, the majority of patients received the combination of grazoprevir/elbasvir (64% of them), followed by the combination of sofosbuvir/ledipasvir (32% of them) with only one patient (4%) receiving the combination of ombitasvir/paritaprevir/ritonavir/dasabubir.

### 3.2. HCV Co-Infection Significantly Impacts on T-Cells Homeostasis

To evaluate the role of HCV co-infection on immune parameters of HIV pathogenesis we compared the phenotypic profile of both CD4 and CD8 T-cells between HIV monoinfected and HIV/HCV coinfected patients before HCV eradication with the new DAAs-based therapy.

**Figure 1:**
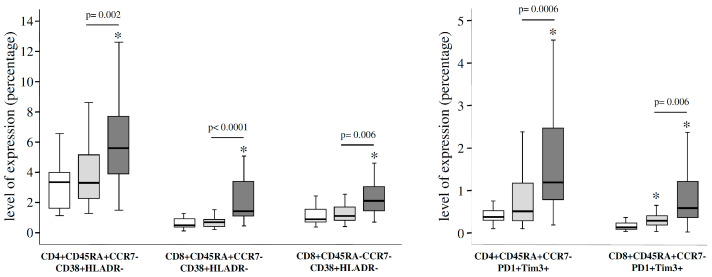
Box-plots showing differences in the level of expression for several CD4 and CD8 T-cell subsets between seronegative controls (white boxes), human immunodeficiency virus (HIV) monoinfected patients (light-grey boxes) and pre-direct acting antivirals (DAAs) HIV/ hepatitis C virus (HCV) coinfected patients (dark-grey boxes). *p*-values for the comparison between monoinfected and coinfected patients are shown. An asterisk (*) indicates significant difference (*p* < 0.05) compared to seronegative controls by Mann-Whitney U test.

We first applied a univariate analysis to compare the expression levels of the different 182 studied T-cell subsets. This analysis showed that 43 unique T-cell subsets were differentially expressed between the different groups of studied individuals. As expected, the majority of these T-cell subsets presented significant differences between SNC individuals and both groups of patients. However, there were a few T-cell subsets significantly different between HIV and pre-DAAs HIV/HVC patients (Supplementary Table S3), within them it is important to highlight different CD4 and CD8 T-cell subsets expressing markers associated to the activation and exhaustion phenomena, with a significantly higher level of expression in the group of pre-DAAs HIV/HCV patients (Figure 1). Then, we used a multivariate general linear model (GLM) analysis to confirm the differences in the expression levels of the different studied T-cell subsets between HIV and pre-DAAs HIV/HCV patients, adjusting by different covariates (age, time since HIV diagnosis, time on ART, CD4 count, percentage of CD4, percentage of CD8). This analysis showed that levels of both exhaustion and activation of effector CD4 T-cells were significantly increased in pre-DAAs HIV/HCV patients compared to HIV patients, in agreement with the results of the univariate analysis (Supplementary Table S4). 

**Figure 2:**
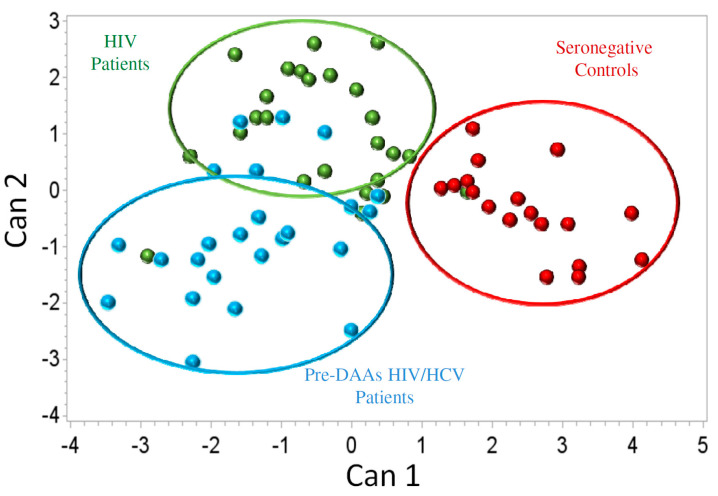
Canonical discriminant analysis comparing the phenotypic profile of T cells between HIV-monoinfected patients (green dots), pre-DAAs HIV/HCV coinfected patients (blue dots) and seronegative controls (red dots).

Additionally, a canonical discriminant analysis (CDA) was carried out to find the variables (T-cell subsets) that maximize the separation between studied groups. A detailed description of the results obtained in this analysis is shown in Supplementary Results S1. Seronegative controls (SNC individuals group) were clearly separated from both groups of HIV-infected patients. However, there was also a good separation between HIV and pre-DAAs HIV/HCV patients. Moreover, HIV patients were closer to seronegative controls than were pre-DAAs HIV/HCV patients (Figure 2). Mean ± SD values for canonical function 1 (Can1) in the different groups of individuals were—2.42 ± 0.85; −0.46 ± 1.05; −1.43 ± 1.06 in seronegative controls, HIV patients and pre-DAAs HIV/HCV patients, respectively. According to the coefficients of the different T-cell subsets in the canonical functions, the T-cell subsets with the highest contribution to discriminate between HIV and pre-DAAs HIV/HCV groups were effector CD4 and effector CD8 T-cells expressing markers associated to activation and exhaustion. Levels of these subsets in pre-DAAs HIV/HCV and in HIV patients are shown in Table 2.

### 3.3. The Degree of Liver Stiffness Significantly Impacts on T-Cells Homeostasis

**Figure 3:**
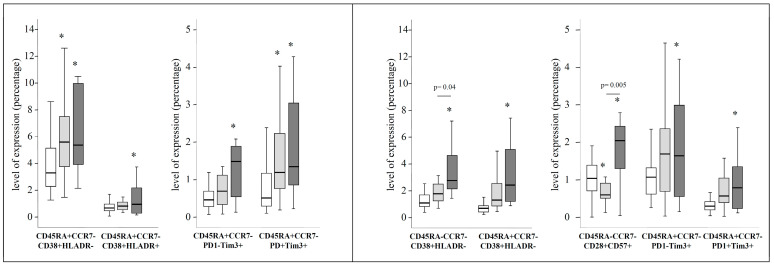
Box-plots showing differences in the level of expression for several CD4 (left graphs) and CD8 (right graphs) T-cell subsets between HIV monoinfected patients (white boxes), pre-DAAs HIV/HCV coinfected patients with liver stiffness (LS) < 7.1 Kpa (light-grey boxes) and in pre-DAAs HIV/HCV coinfected patients with LS ≥ 7.1 KPa (dark-grey boxes). *p*-values for the comparison between HIV/HCV with LS < 7.1 Kpa and LS ≥ 7.1 KPa are shown. An asterisk (*) indicates significant (*p* < 0.05) difference compared to HIV monoinfected patients by Mann-Whitney U test.

The potential impact of liver stiffness (LS) on the levels of the different studied T-cell subsets in the distinct studied group of patients was evaluated. The univariate analysis (Kruskall-Wallis test) revealed that several T-cell subsets, mainly activation and exhaustion of CD4 and CD8 T-cells, presented significant differences when comparing HIV patients, pre-DAAs HIV/HCV patients with LS ≥ 7.1 KPa and pre-DAAs HIV/HCV patients with LS < 7.1 KPa. Interestingly, when compared to HIV patients, the levels of the majority of these T-cell subsets were significantly different only in pre-DAAs HIV/HCV patients with LS ≥ 7.1 KPa (Figure 3).

A multivariate general linear model (GLM) analysis, adjusting by different covariates (age, time since HIV diagnosis, time on ART, CD4 count, percentage of CD4, percentage of CD8), confirmed the influence of liver stiffness on the levels of both activation and exhaustion of effector CD4 and effectors CD8 T-cell subsets. For the majority of T-cell subsets, significant differences with respect to HIV patients were observed only in pre-DAAs HIV/HCV patients with LS ≥ 7.1 KPa (Supplementary Table S5).

**Figure 4:**
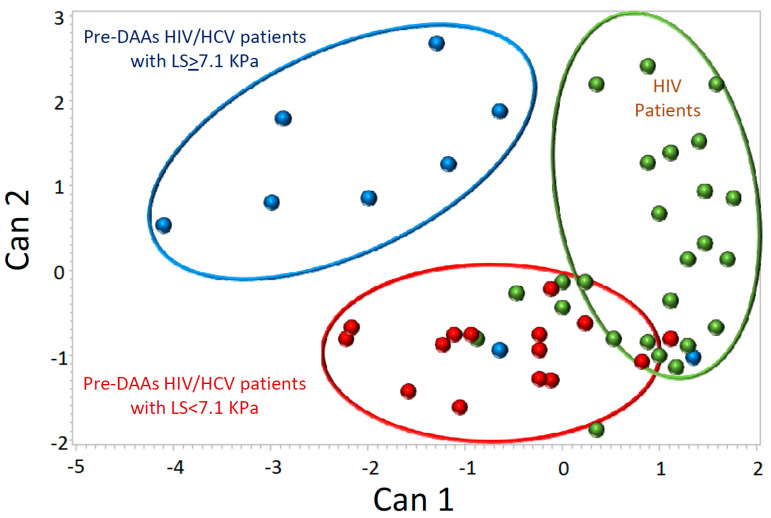
Canonical discriminant analysis comparing the phenotypic profile of T cells between HIV patients (green dots), pre-DAAs HIV/HCV coinfected patients with liver stiffness (LS) < 7.1 Kpa (red dots) and pre-DAAs HIV/HCV coinfected patients with LS ≥ 7.1 Kpa (blue dots).

Lastly, a CDA analysis was performed to ascertain the ability of the studied variables (T-cell subsets) to discriminate between HIV patients and the two groups of pre-DAAs HIV/HCV patients (LS ≥ 7.1 KPa and LS < 7.1 KPa). A detailed description of the results obtained in this analysis is shown in Supplementary Results S2. The results showed that pre-DAAs HIV/HCV patients with LS ≥ 7.1 KPa were clearly separated from the other two groups (HIV monoinfected and pre-DAAs HIV/HCV coinfected with LS < 7.1 KPa) that were close to each other (Figure 4). The T-cell subsets with the greatest weight in separating pre-DAAs HIV/HCV patients with LS ≥ 7.1 KPa from the other two groups were effector CD4 and effector CD8 T-cells expressing markers of activation and exhaustion.

### 3.4. HCV Eradication with DAAs Does Not Completely Revert T-Cells Homeostasis Disturbances

All HIV/HCV patients reached sustained virological response after treatment with DAAs. In parallel, liver enzymes assessed at the time of SVR evaluation returned to normal levels (median(IQR): 18(16–26); 24(20–33); 16(13–25) IU/mL for ALT, AST and GGT respectively). Moreover, liver stiffness was <7.1 KPa in all patients after HCV eradication (after a follow-up median period of 25 months since liver stiffness was not assessed at the time of SVR for the majority of patients). 

**Figure 5:**
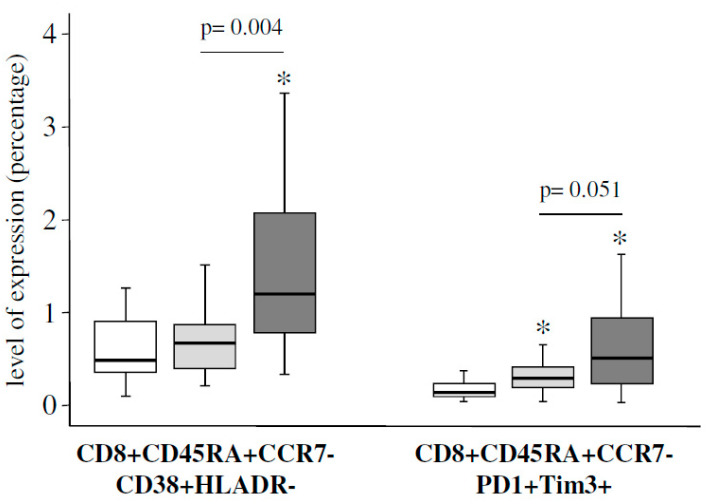
Box-plots showing differences in the level of expression for different CD8 T-cell subsets between seronegative controls (white boxes), HIV monoinfected patients (light-grey boxes) and post-DAAs HIV/HCV coinfected patients (dark-grey boxes). *p*-values for the comparison between monoinfected and coinfected patients are shown. An asterisk (*) indicates significant (*p* < 0.05) difference compared to seronegative controls by Mann-Whitney U test.

**Figure 6:**
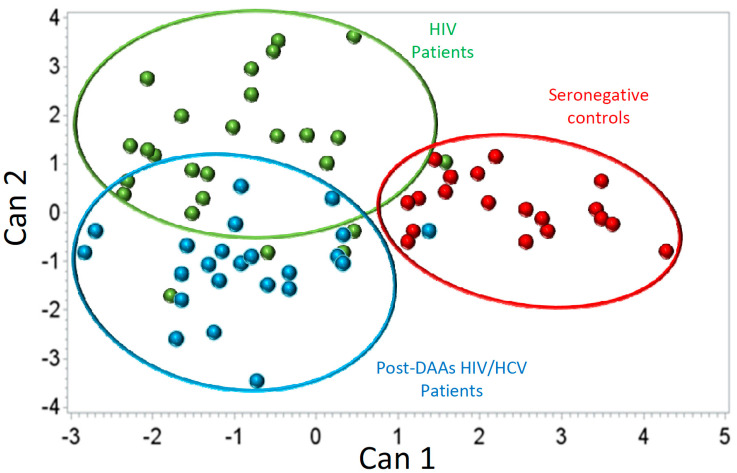
Canonical discriminant analysis comparing the phenotypic profile of T cells between HIV-monoinfected patients (green dots), post-DAAs HIV/HCV coinfected patients (blue dots) and seronegative controls (red dots).

We evaluated to what extent the T-cells homeostasis disturbances observed in pre-DAAs HIV/HCV patients (before HCV eradication) were reverted after HCV eradication (post-DAAs HIV/HCV patients) to values similar to those found in HIV monoinfected patients. We first analyzed the evolution of T-cells subsets that were significantly different in pre-DAAs HIV/HCV patients compared to HIV patients and found that after HCV eradication some of them but not all experienced a significant variation. Thus, exhaustion and activation of effector CD4 T-cells as well as activation of effector memory CD8 T-cells significantly decreased, returning to levels similar to those of HIV patients. However, exhaustion and activation of effector CD8 T-cells did not significantly change after DAAs treatment (Supplementary Table S6). In agreement with this, both activation and exhaustion of effector CD8 T-cells remained increased in post-DAAs HIV/HCV patients compared to HIV monoinfected patients (Figure 5). Moreover, the canonical discriminant analysis showed some degree of separation between HIV monoinfected patients and post-DAAs HIV/HCV patients (Figure 6). A detailed description of the results obtained in this analysis is shown in Supplementary Results S3. Interestingly, the T-cell subsets that most influenced the separation between HIV and post-DAAs HIV/HCV groups were both exhaustion and activation of effector CD8 T-cells, in agreement with the results of the univariate analysis. Levels of these subsets in post-DAAs HIV/HCV and in HIV patients are shown in Table 3.

## 4. Discussion

The main findings of our study are—(a) the presence of HCV coinfection significantly impacts on several immune parameters in patients with HIV infection, especially on T-cells activation and exhaustion; (b) The impact on immune parameters is more marked in HIV/HCV coinfected patients with increased liver stiffness; (c) eradication of HCV with DAAs-based therapy does not completely restore the T-cells homeostasis perturbations observed in HIV/HCV coinfected patients.

Our study shows that coinfection with HCV in HIV-infected patients significantly impacts on T-cells homeostasis, in agreement with previous studies [16,17,18,19,20,21,22,23,24,25,26]; however, our study differs from the previous studies in several aspects—(a) we analyzed a very homogeneous population of HIV patients, all on long-term successful ART and with high CD4 counts; (b) we performed an in-depth characterization of several parameters of T-cells homeostasis; (c) we applied a statistical analysis that enabled us to detect subtle but relevant differences in the profile of T-cells homeostasis between HIV and HIV/HCV patients; (d) the anti-HCV treatment for all included HIV/HCV coinfected patients was the new DAAs-based therapy, IFNα/ribavirin-free-based regimens; thus avoiding the bias of the immunomodulatory effect of IFN-α.

Firstly, we show that in spite of long-term suppression of HIV replication and high CD4 counts, the profile of T-cells homeostasis in the whole population of HIV patients clearly departs from that of seronegative controls, demonstrating that restoration of T-cells homeostasis is no complete even after several years of complete suppression of HIV replication and high CD4 counts, a finding that is in agreement with previous reports [32]. However, our study is the first showing that this impairment of T-cells homeostasis affects to several processes including activation, exhaustion, senescence and apoptosis of both CD4 and CD8 T-cells. 

Secondly, we found that, in the canonical discriminant analysis, HIV/HCV patients formed a cluster clearly separated not only from seronegative controls but also from HIV patients. Moreover, the cluster of HIV/HCV patients was more separated from the cluster of seronegative controls than was the cluster of HIV patients, supporting that HCV coinfection increases the perturbations of T-cells homeostasis associated to HIV infection. Of note, both the canonical and the univariate and multivariate analyses pointed to activation and exhaustion of effector populations of T-cells as the most important to discriminate between HIV/HCV coinfected and HIV monoinfected patients. These results are in agreement with previous studies showing a significant impact of HCV coinfection on T-cells activation [16,17,33,34,35] and/or exhaustion [18,24]. Similar results were found in a recent study by our group employing a cohort of patients with untreated HIV infection, suggesting that in the setting of HIV infection, HCV impacts on T-cells homeostasis independently of the level of HIV replication [26]. 

T-cells activation and exhaustion are important factors in the pathogenesis of HIV disease and thus, the increases we observed in coinfected patients may potentially have relevant clinical implications. Activation of CD8 T-cells has a central role in HIV progression as has been extensively reported [36]. More recently, T-cells exhaustion has been shown as another important factor involved in HIV disease progression, both in cross-sectional [37] and longitudinal [38,39] studies. Interestingly, the increase in expression of exhaustion markers that we found in HIV/HCV coinfected patients was restricted to the effector subset of T-cells, what could impact on the anti-viral activity of these cells because exhaustion of T-cells is a pivotal mechanism of virus escape from immune response in HIV [40] and in HCV [41] infections.

Another interesting finding of our study was the association of the degree of liver stiffness (LS) with perturbations of T-cells homeostasis, an aspect that has been scarcely addressed. Only two previous studies have explored the association between T-cells activation and the degree of LS in HIV/HCV co-infection and have reported an increase in activation in patients with increased values of LS [35,42]. Our study confirms and extends these previous observations, showing perturbations not only in activation but also in exhaustion, which is another important aspect of T-cells homeostasis. Although activation and exhaustion of effector T-cells were the most relevant subsets associated to the degree of LS in our study, the discriminant analysis suggests the existence of subtle perturbations in many other aspects of T-cells homeostasis in HIV/HCV patients with LS ≥ 7.1 KPa, including senescence of effector memory CD8 T-cells and apoptosis of naïve CD4 T-cells. These perturbations of T-cells homeostasis could be involved in the development of liver damage since immune activation and systemic inflammation have been proposed as drivers of hepatic injury [43,44].

Curiously, both activation and exhaustion of effector CD8 T-cells remained significantly increased in HIV/HCV patients after HCV eradication (3 months after the end of DAAs-therapy, SVR) compared to HIV patients. In agreement, the discriminant analysis showed some separation between HIV monoinfected patients and post-DAAs HIV/HCV coinfected patients, supporting that eradication of HCV with DAAs-based therapy is not able to completely revert T-cells homeostasis disturbances, at least in the short term. An explanation that may account for this finding is that normalization of T-cells homeostasis may take longer than the follow up period evaluated in our study (3 months after the end of DAAs-therapy, when sustained virological response, SVR, is evaluated), as has been shown in the context of HIV infection after ART. In fact, several T-cells homeostasis disturbances persisted in the whole population of HIV patients analyzed in our study in spite of several years of ART and complete suppression of HIV replication. Further studies with longer follow-up are needed to ascertain if T-cells homeostasis disturbances associated to HCV coinfection are completely restored over time after HCV eradication with DAAs-based therapy.

There are some controversial issues in our study. First, the relatively small sample size that might preclude detecting more differences associated with the presence of HCV in HIV/HCV coinfected patients. However, the sophisticated statistical analysis that we employed including canonical discriminant analyses was able to overcome this limitation to a large extent since this statistical approach allowed us to detect subtle but important differences in variables that maximize the separation between studied groups. In fact we were able to clearly separate HIV/HCV coinfected patients from HIV monoinfected patients in spite of the relatively small sample size. Second, the interpretation of liver stiffness data should be done carefully because elastography is a technique that can be biased by the presence of necroinflammatory activity in the liver [45,46]. Thus, increased liver stiffness can reflect either fibrosis or necroinflammatory activity, being both conditions indicative of liver damage. Interestingly, in our study those HIV/HCV patients with liver stiffness ≥7.1 KPa did also have higher values of the hepatic enzymes AST and ALT suggesting a higher degree of necroinflammatory activity. However, after HCV eradication with DAAs-based therapy (3 months after the end of DAAs-therapy, when sustained virological response, SVR, is evaluated) normal levels of the liver enzymes were observed. Furthermore, after a median follow-up of 25 months after the end of DAA-based therapy, liver stiffness was <7.1 KPa in all HIV/HCV coinfected patients. 

In summary, our study is the first addressing the impact of HCV co-infection on several aspects of T-cells homeostasis in a very homogenous population of HIV-infected patients. We used a robust methodology, both experimentally and statistically, that allowed us to address not only a broad spectrum of T-cell homeostasis parameters but also allowed us to detect any subtle difference in these parameters between the two study groups. We were able to identify the T-cell subsets (mainly effector CD4 and effector CD8 T-cells expressing markers associated to activation and exhaustion) with the highest contribution to discriminate between HIV monoinfected and HIV/HCV coinfected groups. Overall, our findings show a significant impact of HCV on several aspects of immune pathogenesis of HIV disease, especially in those patients with increased liver stiffness but this impact of HCV coinfection was not fully reversed (with activation and exhaustion of effector CD8 T-cells remaining significantly increased) after eradication of HCV with DAAs-based therapy. Thus, in the management of patients with HIV infection, particular attention should be paid to HIV/HCV coinfected patients, since there may be active pathogenic mechanisms despite the control of both HIV and HCV replication, which prompts the necessity to explore on other mechanisms underlying the immune damage observed.

## Figures and Tables

**Table 1:** Characteristics at baseline of patients included in the study.

Characteristic	HIV Group (*n* = 25)	HIV/HCV Group (*n* = 25)	*p*-Value
Age (years)	48 (42–55)	44 (39–48)	0.11
Gender (% of males)	88%	100%	0.24
Time since HIV diagnosis (years)	9 (6–14)	7 (2–10)	0.07
Time since HCV diagnosis (years)	NA	2 (1.5–5)	NA
Time on ART (years)	5 (3.5–7.5)	4 (2–9)	0.63
CD4 count (cells/μL)	816 (605–992)	735 (577–902)	0.31
Ratio CD4/CD8	0.84 (0.56–1.34)	0.75 (0.60–1.09)	0.49
ALT level (IU/L)	32 (24–37)	74 (49–162)	**<0.0001**
AST level (IU/L)	27 (22–31)	58 (37–122)	**<0.0001**
GGT level (IU/L)	36 (22–54)	55 (25–119)	0.06
Total cholesterol level (mg/dL)	194 (162–227)	157 (125–177)	**<0.0001**
HDL level (mg/dL)	42 (37–50)	46 (33–50)	0.815
LDL level (mg/dL)	119 (93–146)	85 (71–104)	**0.001**
HCV-RNA (log copies/mL)	NA	6.1 (5.8–6.4)	NA
HIV transmission route (%)			1
Sexual	100%	96%	
Parenteral	0%	4%	
HCV genotype (%)			NA
1a	NA	60%	
1b	NA	12%	
4	NA	28%	
Liver stiffness (measured by elastography) (%)			NA
<7.1 KPa	NA	60%	
≥7.1 KPa	NA	40%	
DAA regimen			
GZR/EBR	NA	64%	
SOF/LDV	NA	32%	
OBV/PTV/r/DSV	NA	4%	

ART: antiretroviral therapy; ALT: alanine aminotransferase; AST: aspartate aminotransferase; GGT: gamma glutamil transferase; HDL: high density lipoprotein; LDL: low density lipoprotein; HCV: hepatitis C virus. Data for continuous variables are given as median (interquartile range). Significant *p*-values are in bold. NA: not applicable; KPa: kilopascals. DAAs: direct-acting antivirals; GZR: grazoprevir; EBR: elbasvir; SOF: sofosbuvir; LDV: ledipasvir; OBV: ombitasvir; PTV: paritaprevir, r: ritonavir; DSV: dasabuvir

**Table 2:** Levels of different T-cell subsets in HIV/HCV coinfected patients before DAAs treatment (pre-DAAs) compared to HIV monoinfected patients.

T-Cell Subsets	Level of T-Cell Subsets Median [IQR]		*p*-Value *
	pre-DAAs HIV/HCV Coinfected Patients	HIV Monoinfected Patients	
CD4+CD45RA+CCR7-PD1-TIM3+	0.91 (0.45–1.57)	0.46 (0.27–0.74)	0.076
CD4+CD45RA+CCR7-CD38+HLADR-	5.59 (3.84–8.83)	3.29 (2.19–0.47)	0.004
CD8+CD45RA+CCR7-PD1+TIM3+	0.59 (0.31–1.25)	0.29 (0.18–0.44)	0.006
CD8+CD45RA+CCR7-CD38+HLADR-	1.41 (1.01–3.66)	0.67 (0.39–0.91)	<0.0001

* *p*-values of comparison between groups by Mann-Whitney U test.

**Table 3:** Levels of different T-cell subsets in HIV/HCV coinfected patients after DAAs treatment (post-DAAs) compared to HIV monoinfected patients.

T-Cell Subsets	Level of T-Cell Subsets Median [IQR]		*p*-Value *
	post-DAAs HIV/HCV Coinfected Patients	HIV Monoinfected Patients	
CD8+CD45RA+CCR7-PD1+TIM3+	0.51 (0.23–0.99)	0.29 (0.18–0.44)	0.051
CD8+CD45RA+CCR7-CD38+HLADR-	1.20 (0.77–2.51)	0.67 (0.39–1.58)	0.003

* *p*-values of comparison between groups by Mann-Whitney U test.

## Supplementary Materials

The following are available online at https://www.mdpi.com/2077-0383/9/9/2978/s1, Supplementary Methods; Supplementary Figure S1: Representative example of flow cytometry analysis. Dot-plots in the row (A) show the gating strategy employed to gate CD4+ and CD8+ T cells. The other two rows (B and C) show the phenotype of CD4+ and CD8+ T cells according to the different surface markers included in the staining panel to define different aspects of T cell homeostasis. Supplementary Figure S2: Fluorescence minus one (FMO) controls employed to set the gates defining positive versus negative population of cells for those markers with low and/or continuous expression. Gates for each marker were set on CD3+ T cells. Supplementary Results S1: Results of the PLS model and canonical discriminant analysis carried out to discriminate between seronegative controls (SNC), patients monoinfected with HIV (HIV) and patients coinfected with HIV and HCV before treatment with DAAs (pre-DAAs HIV/HCV). Supplementary Results S2: Results of the PLS model and canonical discriminant analysis carried out to discriminate between patients monoinfected with HIV (HIV), patients coinfected with HIV and HCV before treatment with DAAs and liver stiffness (LS) values < 7.1 KPa (pre-DAAs HIV/HCV with KPa < 7.1) and patients coinfected with HIV and HCV before treatment with DAAs and liver stiffness (LS) values > 7.1 KPa (pre-DAAs HIV/HCV with KPa > 7.1). Supplementary Results S3: Results of the PLS model and canonical discriminant analysis carried out to discriminate between seronegative controls (SNC), patients monoinfected with HIV (HIV) and patients coinfected with HIV and HCV after treatment with DAAs (post-DAAs HIV/HCV) Supplementary Table S1: Monoclonal antibodies and fluorochromes used in the study. Supplementary Table S2: List of T-cell subsets analyzed with their level of expression (median and interquartile range) in the different groups of individuals included in the study. Supplementary Table S3: T-cell subsets presenting statistically significant differences (in the univariate analysis) between the study groups (HIV group, pre-DAAs HIV/HCV group and SNC group/seronegative controls). In bold, T-cell subsets presenting significant differences (*p* < 0.017 after adjusting for multiple comparisons) between HIV and pre-DAAs HIV/HCV groups. Supplementary Table S4: Multivariate Analysis (GLM): CD4 T-cell subsets with significant differences (*p* < 0.05), in their expression levels, between HIV and pre-DAAs HIV/HCV patients, after adjusting for several covariates. Table shows the coefficient and the adjusted p-value for the factor “patients group (HIV = 0, HIV/HCV = 1)” in the GLM analysis for each of the studied T-cell subsets. Supplementary Table S5: Multivariate Analysis (GLM): T-cell subsets with significant differences (*p* < 0.05), in their expression levels, between HIV patients, pre-DAAs HIV/HCV patients with liver stiffness (LS) > 7.1 KPa and pre-DAAs HIV/HCV patients with LS < 7.1 KPa, after adjusting for several covariates. Table shows the coefficients in the GLM analysis and the adjusted *p*-value for each of the studied T-cell subsets for pre-DAAs HIV/HCV patients with LS > 7.1 KPa and for pre-DAAs HIV/HCV patients with LS < 7.1 KPa, taking as reference the HIV group. Supplementary Table S6: Levels of different T-cell subsets in HIV/HCV coinfected patients before DAAs treatment (pre-DAAs) and after DAAs treatment (post-DAAs). In bold, T-cell subsets presenting significant differences.
