# Supplementary material for: Both HCV Infection and Elevated Liver Stiffness Significantly Impacts on Several Parameters of T-Cells Homeostasis in HIV-Infected Patients
Source: J Clin Med. 2020 Sep 15;9(9):2978. doi: 10.3390/jcm9092978 (PMC7564456; doi:10.3390/jcm9092978)
Supplement: Supplementary file 1 [file jcm-09-02978-s001.zip › jcm-924735 suppl/2. Figure S1.pdf]

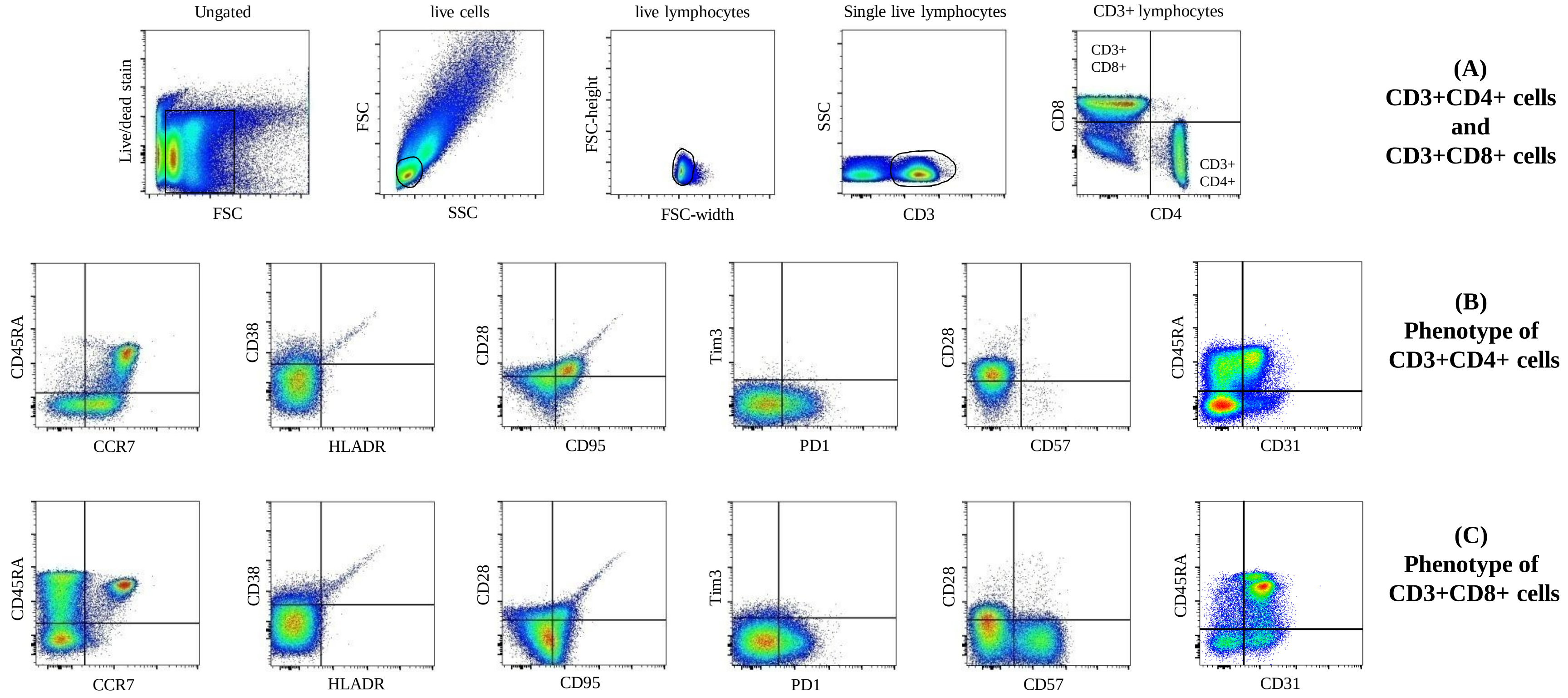

Supplementary Figure S1. Representative example of flow cytometry analysis. Dot-plots in the row **(A)** show the gating strategy employed to gate CD4+ and CD8+ T cells. The other two rows (**B** and **C**) show the phenotype of CD4+ and CD8+ T cells according to the different surface markers included in the staining panel to define different aspects of T cell homeostasis
