# Supplementary material for: Both HCV Infection and Elevated Liver Stiffness Significantly Impacts on Several Parameters of T-Cells Homeostasis in HIV-Infected Patients
Source: J Clin Med. 2020 Sep 15;9(9):2978. doi: 10.3390/jcm9092978 (PMC7564456; doi:10.3390/jcm9092978)
Supplement: Supplementary file 1 [file jcm-09-02978-s001.zip › jcm-924735 suppl/3. Figure S2.pdf]

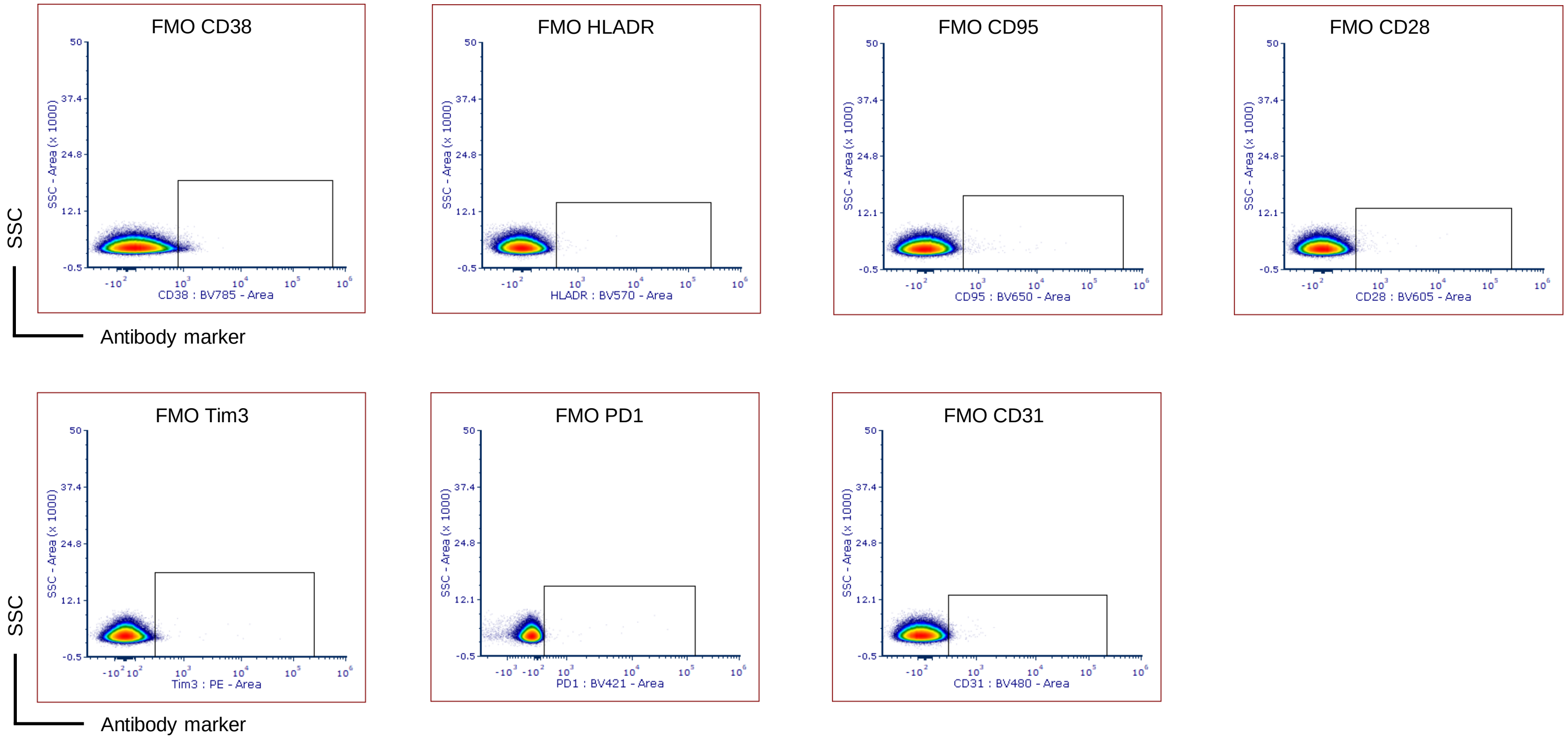

Supplementary Figure S2. Fluoresecence minus one (FMO) controls employed to set the gates defining positive versus negative population of cells for those markers with low and/or continuous expression. Gates for each marker were set on CD3+ T cells.
