# Supplementary material for: Both HCV Infection and Elevated Liver Stiffness Significantly Impacts on Several Parameters of T-Cells Homeostasis in HIV-Infected Patients
Source: J Clin Med. 2020 Sep 15;9(9):2978. doi: 10.3390/jcm9092978 (PMC7564456; doi:10.3390/jcm9092978)
Supplement: Supplementary file 1 [file jcm-09-02978-s001.zip › jcm-924735 suppl/1. Supplementary Methods.doc]

Supplementary Methods

Staining Procedure and Flow Cytometry Analysis

A million of PBMCs were stained with a panel of thirteen different monoclonal antibodies (plus a viability dye) that in combination defined a total of 182 unique T cell subsets. After staining, cells were acquired using an SP 6800 Spectral flow cytometer (Sony) and a minimum of 200,000 events were acquired for further analysis. Data analysis was performed using FlowJo (Treestar, San Carlos, CA). An initial gating was applied to exclude dead cells using the live/dead viability dye, then lymphocytes were gated based on forward (FSC) and side (SSC) scatter, and finally single cells were selected using FSC-width *versus* FSC-height. Starting from the population of single live lymphocytes, a gate was placed to select T cells (CD3+ lymphocytes) and then CD3+CD4+ or CD3+CD8+ T cells were selected for further analysis. Fluorescence minus one (FMO) controls were used for those markers with low and/or continuous expression to set the gate defining the positive versus negative population (**Supplementary Figure S2**).

Discriminant Models

Discriminant models were generated to separate between different groups of subjects (HIV patients, HIV/HCV patients and SNC individuals) and between different timepoints (pre and post-DAA therapy in HIV/HCV patients) with the aim to find out which T-cell subsets were more relevant to discriminate between different groups. For this purpose, first a partial least square (PLS) regression was employed to reduce the dimensionality of data and generate a set of uncorrelated PLS components that were subsequently employed as input variables to perform a canonical discriminant analysis.

Partial Least Squares (PLS) Regression

It is a technique that reduces the number of predictor variables to a smaller set of uncorrelated components and performs least squares regression on these components, instead of on the original data. PLS regression is especially useful when the predictor variables are highly collinear, or when there are far more predictor variables than observations and ordinary least-squares regression either produces coefficients with high standard errors or fails completely.

In PLS, the observable (measured) predictor variables are grouped together to generate a minimum set of latent variables (LV) not directly observed but rather inferred through a linear combination of the original set of observable variables. This method reduces the dimensionality of data [1, 2].

In this study, PLS was used as a tool for modeling the different groups or classes of subjects included in the study (seronegative controls -SNC individuals-, HIV mono-infected patients, HIV/HCV co-infected patients), using the set of different immunological variables (182 in total) as the predictor variables and the subject´s group as the response (predicted) categorical variable. In the different analyses, PLS was used to make a predictor variables selection and reduce the original 182 to a smaller number. The parameter employed, called variable influence on projection, VIP, summarizes the importance of the predictor (X) variables. A variable with a VIP score close to or greater than 0.8 (Wold Criterium) can be considered important in the given model [1, 2]. Variables with VIP scores significantly less than 0.8 are less important and might be good candidates for exclusion from the model. The procedure was as follows:

1. Obtain the number of latent variables that minimized the sum of squares of the expected residuals after cross validation using the PRESS procedure.
2. Remove those samples with standardized residual (in absolute value) greater than 2.5 and with Q and T2 values significantly higher than a threshold at 99% confidence level.
3. Repeat steps (a) and (b) until there were no outliers.

Canonical Discriminant Analysis (Fisher’s Method)

It is a multivariate technique whose main objective is to separate populations. That is, try to find qualitative explanations to describe why populations are different. It is therefore essentially a descriptive technique [3].

The populations are separated by canonical discriminant functions y_i_ (i=1,…,m) as a linear combination of discriminant variables x_j_ (j=1,…,p). The canonical variables must be less than the number of discriminant variables. This approach has clear advantages in order to be able to graphically describe populations if we are left with 3 or less canonical variables. The issues to be resolved are: How do we obtain the coefficients a_i j_?. How many canonical variables are necessary? Can the canonical variables be interpreted? [3].

The coefficients have to be determined in such a way that the average of the first canonical function for each class is as different as possible. So it is about maximizing differences between groups and minimizing differences within groups. For this we help ourselves with two matrices: one measures the variables variability between groups, and another measures the variables variability intra groups.

After the first canonical function has been obtained, we will look for the second canonical function in such a way that all the lines perpendicular to the first canonical function are those that generate the plane of greatest difference (or distance) between the means of the groups.

The following tools are available to help interpret the canonical variables [3]:

- Standardized coefficients serve to determine which variables contribute most to determining the values of the canonical function. This is achieved by simply examining the magnitude of the standardized coefficients (ignoring the sign). The greater the magnitude, the greater the contribution of the variable.
- The structure coefficients are simple bivariate correlations that are not affected by the relation of the other variables as is the case with the standardized coefficients.

In the paper, PLS has been combined with Canonical discriminant analysis. First, uncorrelated PLS components have been obtained and subsequently these have served as input variables to perform canonical discriminant analysis. The canonical discriminant analysis works better if the input variables are uncorrelated. This has allowed to represent graphically in a space of low dimension the observations (patients) characterizing them by the group to which they belong.

Software Employed

SAS software has been used to calculate the Canonical Discriminant Analysis (Proc Candisc), and PLS Regresion (Proc PLS) [4]. In addition, macros of own elaboration have been made to generate sequence of input-output variables in the models.

References

1. Tennenhaus, M. (1998): La règression PLS:thèorie et pratique. Editions technip. Paris.
2. Wold, S. (1994) PLS for Multivariate Linear Modeling. Chemometric methods in Molecular Design, Methdos and Principies in Medicinal Chemistry. Ed. Van de Waterbeemd, 195-218. Weinhei, Germany, Verlag-Chemie.
3. Rao, C. R. (1973). Linear Statistical Inference and Its Applications. 2nd ed. New York: John Wiley & Sons.
4. Little R.C., Freund, R. J., Spector P C. (2002). SAS System for Linear Models, fourth Edition. SAS series in Statistica appliations. Sas Institute, Cary, North Caroline. USA.
