# Supplementary material for: Both HCV Infection and Elevated Liver Stiffness Significantly Impacts on Several Parameters of T-Cells Homeostasis in HIV-Infected Patients
Source: J Clin Med. 2020 Sep 15;9(9):2978. doi: 10.3390/jcm9092978 (PMC7564456; doi:10.3390/jcm9092978)
Supplement: Supplementary file 1 [file jcm-09-02978-s001.zip › jcm-924735 suppl/10. Table S4.doc]

**Table S4.** Multivariate Analysis (GLM): CD4 T-cell subsets with significant differences (p<0.05), in their expression levels, between HIV and pre-DAAs HIV/HCV patients, after adjusting for several covariates. Table shows the coefficient and the adjusted p-value for the factor “patients group (HIV=0, HIV/HCV=1)” in the GLM analysis for each of the studied T-cell subsets.

| **CD4 T-cell subsets** | **Biological process** | **Coefficient in the GLM analysis^1^** | **Adjusted**  **p-value^2^** |
| --- | --- | --- | --- |
| CD4+CD45RA+CCR7-PD1-Tim3+ | Exhaustion | 0.7907 | 0.0019 |
| CD4+CD45RA+CCR7-PD1+Tim3+ | Exhaustion | 0.9645 | 0.0020 |
| CD4+CD45RA+CCR7-CD38+HLADR- | Activation | 0.8216 | 0.0066 |
| CD4+CD45RA+CCR7-CD28-CD57+ | Senescence | -0.7653 | 0.0085 |
| CD4+CD45RA+CCR7-CD28+CD57- | Senescence | 0.5848 | 0.0488 |

**(1)**: a positive coefficient means higher values for the T-cell subset in HIV/HCV patients; a negative coefficient means lower levels of the T-cell subset in HIV/HCV patients

**(2)**: adjusted for age, time since HIV diagnosis, time on ART, CD4 count, CD4 percentage, CD8 percentage

**GLM**: General Linear Model .
