# Supplementary material for: Both HCV Infection and Elevated Liver Stiffness Significantly Impacts on Several Parameters of T-Cells Homeostasis in HIV-Infected Patients
Source: J Clin Med. 2020 Sep 15;9(9):2978. doi: 10.3390/jcm9092978 (PMC7564456; doi:10.3390/jcm9092978)
Supplement: Supplementary file 1 [file jcm-09-02978-s001.zip › jcm-924735 suppl/11. Table S5.doc]

**Table S5.** Multivariate Analysis (GLM): T-cell subsets with significant differences (p<0.05), in their expression levels, between HIV patients, pre-DAAs HIV/HCV patients with liver stiffness (LS)≥7.1 KPa, and pre-DAAs HIV/HCV patients with LS<7.1 KPa, after adjusting for several covariates. Table shows the coefficients in the GLM analysis and the adjusted p-value for each of the studied T-cell subsets for pre-DAAs HIV/HCV patients with LS≥7.1 KPa and for pre-DAAs HIV/HCV patients with LS<7.1 KPa, taking as reference the HIV group.

| **T-cell subset** | **Biological process** | **Coefficient in the GLM analysis^1^ for pre-DAAs HIV/HCV patients with LS≥7.1 KPa** | **Adjusted**  **p-value^2^** | **Coefficient in the GLM analysis^1^ for pre-DAAs HIV/HCV patients with LS<7.1 KPa** | **Adjusted**  **p-value^2^** |
| --- | --- | --- | --- | --- | --- |
| CD4+CD45RA+CCR7-CD38+HLADR+ | Activation | 1.1416 | 0.0029 | 0.1932 | 0.5252 |
| CD4+CD45RA+CCR7-  PD1-Tim3+ | Exhaustion | 1.0810 | 0.0008 | 0.4894 | 0.0567 |
| CD4+CD45RA+CCR7-  PD1+Tim3+ | Exhaustion | 1.2168 | 0.0019 | 0.8272 | 0.0099 |
| CD8+CD45RA+CCR7-CD38+HLADR- | Activation | 1.0582 | 0.0037 | 0.2883 | 0.3210 |
| CD8+CD45RA+CCR7-  PD1-Tim3+ | Exhaustion | 1.0137 | 0.0084 | 0.1244 | 0.6866 |
| CD8+CD45RA+CCR7-  PD1+Tim3+ | Exhaustion | 1.0829 | 0.0058 | 0.4900 | 0.1226 |

**(1)**: a positive coefficient means higher values for the T-cell subset in HIV/HCV patients compared to HIV patients

**(2)**: adjusted for age, time since HIV diagnosis, time on ART, CD4 count, CD4 percentage, CD8 percentage

**GLM**: General Linear Model.
