# Supplementary material for: Both HCV Infection and Elevated Liver Stiffness Significantly Impacts on Several Parameters of T-Cells Homeostasis in HIV-Infected Patients
Source: J Clin Med. 2020 Sep 15;9(9):2978. doi: 10.3390/jcm9092978 (PMC7564456; doi:10.3390/jcm9092978)
Supplement: Supplementary file 1 [file jcm-09-02978-s001.zip › jcm-924735 suppl/12. Table S6.doc]

**Table S6.** Levels of different T-cell subsets in HIV/HCV coinfected patients before DAAs treatment (pre-DAAs) and after DAAs treatment (post-DAAs). In bold, T-cell subsets presenting significant differences.

| **T-cell subsets** | **Level of T-cell subsets on HIV/HCV coinfected patients. Median [IQR]** | | **p-value*** |
| --- | --- | --- | --- |
|  | **pre-DAAs** | **post-DAAs** |  |
| **CD4+CD45RA+CCR7-PD1+TIM3+** | **1.19 [0.76 – 2.76]** | **0.70 [0.48 – 1.22]** | **0.023** |
| **CD4+CD45RA+CCR7-CD38+HLADR-** | **5.59 [3.84 – 8.83]** | **3.60 [2.98 – 5.84]** | **0.040** |
| CD8+CD45RA+CCR7-PD1+TIM3+ | 0.59 [0.31 – 1.25] | 0.51 [0.23 -0.99] | 0.461 |
| CD8+CD45RA+CCR7-CD38+HLADR- | 1.41 [1.01 – 3.66] | 1.20 [0.77 – 2.51] | 0.162 |
| **CD8+CD45RA-CCR7-CD38+HLADR-** | **2.13 [1.47 – 3.06]** | **1.47 [0.93 – 2.09]** | **0.016** |

* P-values of comparison between groups by Mann-Whitney U test.
