# Supplementary material for: Both HCV Infection and Elevated Liver Stiffness Significantly Impacts on Several Parameters of T-Cells Homeostasis in HIV-Infected Patients
Source: J Clin Med. 2020 Sep 15;9(9):2978. doi: 10.3390/jcm9092978 (PMC7564456; doi:10.3390/jcm9092978)
Supplement: Supplementary file 1 [file jcm-09-02978-s001.zip › jcm-924735 suppl/5. Results S2.doc]

**Supplementary Results S2**

*Results of the PLS model and canonical discriminant analysis carried out to discriminate between patients monoinfected with HIV (HIV), patients coinfected with HIV and HCV before treatment with DAAs and liver stiffness (LS) values<7.1 KPa (pre-DAAs HIV/HCV with KPa<7.1) and patients coinfected with HIV and HCV before treatment with DAAs and liver stiffness (LS) values≥7.1 KPa (pre-DAAs HIV/HCV with KPa≥7.1)*

Using the 182 T-cells subsets included in the study, a PLS model was first generated to reduce the dimensionality of data. The PLS model selected fifteen different T-cells subsets based on the parameter called variable influence on projection (VIP) that summarizes the importance of the predictor variable on the model taking into account the amount of explained variance of the predicted (response) variable. Then the PLS model was built using the selected set of predictor variables grouped together to generate a minimum set of latent variables (LV). Each LV is a linear combination of the original set of observable variables (T-cells subsets). **Table 1** shows the coefficients of each T-cells subset selected in each of the three latent variables (LV) included in the PLS model.

**Table 1.** Coefficients of the T-cells subsets in each of the latent variables of the PLS model. The absolute value of the coefficient gives a rough estimate of the weight of the T-cells subset in the LV.

|  | Coefficients in the LV of the PLS model | |
| --- | --- | --- |
| **T-cells subsets** | **LV1** | **LV2** |
| CD8+CD45RA+CCR7+CD38-HLADR+ | 0.11672 | 0.43571 |
| CD4+CD45RA+CCR7+CD38-HLADR+ | 0.11513 | 0.44866 |
| CD8+ CD28+ CD57+ | -0.01646 | 0.48179 |
| CD8+CD45RA-CCR7-CD28+CD57+ | -0.05494 | 0.45303 |
| CD8+CD45RA-CCR7-CD28-CD57+ | -0.15209 | 0.30157 |
| CD4+CD45RA+CCR7-CD38+HLADR+ | -0.22607 | 0.14184 |
| CD8+CD45RA+CCR7-PD1-Tim3+ | -0.24110 | 0.08793 |
| CD8+CD45RA+CCR7-PD1+Tim3+ | -0.32052 | -0.02523 |
| CD8+CD45RA+CCR7-CD38+HLADR- | -0.32943 | -0.02501 |
| CD4+ CD45RA+CCR7-CD38+HLADR- | -0.36386 | 0.05534 |
| CD4+CD45RA+CCR7-PD1+Tim3+ | -0.40044 | -0.01606 |
| CD8+CD45RA+CCR7+CD38-HLADR+ | 0.11672 | 0.43571 |
| CD4+CD45RA+CCR7+CD38-HLADR+ | 0.11513 | 0.44866 |
| CD8+ CD28+ CD57+ | -0.01646 | 0.48179 |
| CD8+CD45RA-CCR7-CD28+CD57+ | -0.05494 | 0.45303 |

Next, a canonical discriminant model was built using the three latent variables of the PLS model to further reduce the dimensionality of data without losing information. This model selected two canonical functions (Can) as a lineal combination of the latent variables (LV) of the PLS model. **Table 2** shows the coefficients of each latent variable in each canonical function.

**Table 2.** Raw canonical coefficients for each LV in the canonical functions (Can) of the discriminant model.

|  | **Raw Canonical Coefficients** | |
| --- | --- | --- |
| **Variable** | **Can 1** | **Can 2** |
| **LV1** | 0.6791722973 | -.0894494758 |
| **LV2** | 0.1139639688 | 0.6198884606 |

Using the coefficients of each T-cells subset in the LV of the PLS model and the raw canonical coefficients of each LV in the canonical functions, the coefficients of the T-cells subsets in each canonical function were calculated. **Table 3** shows these coefficients.

**Table 3.** Coefficients of the T-cells subsets in each of the canonical functions (Can) of the discriminant canonical model. The absolute value of the coefficient gives a rough estimate of the weight of the T-cells subset in the canonical function.

| **T-cells subsets** | **Can 1** | **Can 2** |
| --- | --- | --- |
| CD4+CD45RA+CCR7-CD38-HLADR- | 0.26420 | 0.46937 |
| CD8+CD45RA+CCR7-CD38-HLADR- | 0.20227 | 0.53986 |
| CD4+CD45RA+CCR7+CD95+CD28- | 0.19300 | 0.80424 |
| CD4+CD45RA+CCR7+CD28-CD57+ | 0.17139 | 0.76916 |
| CD4+CD45RA+CCR7+CD38- | 0.12933 | 1.05825 |
| CD8+CD45RA+CCR7+CD38- | 0.12893 | 1.04516 |
| CD8+ CD28+ CD57+ | 0.04373 | 1.10315 |
| CD8+CD45RA-CCR7-CD28+CD57+ | 0.01431 | 1.07783 |
| CD8+CD45RA-CCR7-CD28-CD57+ | -0.06892 | 0.93506 |
| CD4+CD45RA+CCR7-CD38+HLADR+ | -0.13738 | 0.78195 |
| CD8+CD45RA+CCR7-PD1-Tim3+ | -0.15373 | 0.72938 |
| CD8+CD45RA+CCR7-PD1+Tim3+ | -0.22057 | 0.62333 |
| CD8+CD45RA+CCR7-CD38+HLADR- | -0.22659 | 0.62435 |
| CD4+ CD45RA+CCR7-CD38+HLADR- | -0.24082 | 0.70777 |
| CD4+CD45RA+CCR7-PD1+Tim3+ | -0.27380 | 0.63965 |

Lastly, values for each canonical function of the model were calculated for each study individual and the mean values were obtained for the different study groups (SNC, HIV and pre-DAAs HIV/HCV). Means for each canonical function are shown in **Table 4.**

**Table 4.** Mean values obtained in each of the study groups for the different canonical (Can) functions.

| **Study Group** | **Can 1** | **Can 2** |
| --- | --- | --- |
| Pre-DAAs HIV/HCV with KPa≥7.1 | -1.545 | 0.897 |
| Pre-DAAs HIV/HCV with KPa<7.1 | -0.573 | -0.894 |
| HIV | 0.900 | 0.213 |
