# Supplementary material for: Both HCV Infection and Elevated Liver Stiffness Significantly Impacts on Several Parameters of T-Cells Homeostasis in HIV-Infected Patients
Source: J Clin Med. 2020 Sep 15;9(9):2978. doi: 10.3390/jcm9092978 (PMC7564456; doi:10.3390/jcm9092978)
Supplement: Supplementary file 1 [file jcm-09-02978-s001.zip › jcm-924735 suppl/7. Table S1.doc]

**Table S1.** Monoclonal antibodies and fluorochromes used in the study.

| **Antibody** | **Fluorochrome** | **Clone** | **Provider** |
| --- | --- | --- | --- |
| CD3 | BV711 | UCHT1 | Biolegend |
| CD4 | PECy5 | RPA-T4 | Biolegend |
| CD8 | PercPCy5.5 | RPA-T8 | Biolegend |
| CD38 | BV785 | HIT2 | Biolegend |
| HLA-DR | BV570 | L243 | Biolegend |
| CCR7 | PE/DAZZLE | G043H7 | Biolegend |
| CD45RA | AF488 | HI100 | Biolegend |
| PD1 | BV421 | EH12-2H7 | Biolegend |
| Tim3 | PE | F38-2E2 | Biolegend |
| CD95 | BV650 | DX2 | Biolegend |
| CD28 | BV605 | CD28.2 | BD Biosciences |
| CD57 | PECy7 | HNK-1 | Biolegend |
| CD31 | BV480 | WM59 | BD Biosciences |
