# Supplementary material for: Both HCV Infection and Elevated Liver Stiffness Significantly Impacts on Several Parameters of T-Cells Homeostasis in HIV-Infected Patients
Source: J Clin Med. 2020 Sep 15;9(9):2978. doi: 10.3390/jcm9092978 (PMC7564456; doi:10.3390/jcm9092978)
Supplement: Supplementary file 1 [file jcm-09-02978-s001.zip › jcm-924735 suppl/9. Table S3.doc]

**Table S3.** T-cell subsets presenting statistically significant differences (in the univariate analysis) between the study groups (HIV group, pre-DAAs HIV/HCV group, and SNC group/seronegative controls). In bold, T-cell subsets presenting significant differences (p<0.017 after adjusting for multiple comparisons) between HIV and pre-DAAs HIV/HCV groups.

| **CD4 T-cell subsets** | **Biological process** | **p-value  (all groups)** | **p-value  (SNC vs. HIV)** | **p-value ( SNC vs. pre-DAAs HIV/HCV)** | **p-value ( HIV vs. pre-DAAs HIV/HCV)** |
| --- | --- | --- | --- | --- | --- |
| CD4+PD1+ Tim3+ | Exhaustion | 0.000365 | 0.00413 | 0.00018 | 0.17423 |
| CD4+PD1+ Tim3- | Exhaustion | 0.000857 | 0.00612 | 0.00021 | 0.54750 |
| CD4+PD1- Tim3- | Exhaustion | 0.002030 | 0.00532 | 0.00085 | 0.74151 |
| **CD4+CD45RA+CCR7-PD1+Tim3+** | **Exhaustion** | 0.000094 | 0.27758 | 0.00016 | **0.00057** |
| CD4+CD45RA+CCR7-PD1+Tim3- | Exhaustion | 0.001205 | 0.01498 | 0.00019 | 0.39323 |
| CD4+CD45RA+CCR7-PD1-Tim3- | Exhaustion | 0.000216 | 0.01238 | 0.00002 | 0.21789 |
| CD4+CD45RA+CCR7+PD1+Tim3+ | Exhaustion | 0.004363 | 0.00725 | 0.00297 | 0.43736 |
| CD4+CD45RA-CCR7+PD1+Tim3+ | Exhaustion | 0.000195 | 0.00204 | 0.00004 | 0.86891 |
| CD4+CD45RA-CCR7+PD1+Tim3- | Exhaustion | 0.000005 | 0.00009 | 0.00000 | 0.30376 |
| CD4+CD45RA-CCR7+PD1-Tim3- | Exhaustion | 0.000004 | 0.00007 | 0.00000 | 0.32708 |
| CD4+CD45RA-CCR7-PD1+Tim3+ | Exhaustion | 0.006551 | 0.15656 | 0.00127 | 0.09903 |
| CD4+CD45RA-CCR7-PD1+Tim3- | Exhaustion | 0.000003 | 0.00105 | 0.00000 | 0.10513 |
| CD4+CD45RA-CCR7-PD1-Tim3- | Exhaustion | 0.000003 | 0.00075 | 0.00000 | 0.10516 |
| **CD4+ CD45RA+CCR7-CD38+HLADR-** | **Activation** | 0.000714 | 0.46481 | 0.00079 | **0.00217** |
| CD4+CD45RA+CCR7-CD38-HLADR- | Activation | 0.001742 | 0.40425 | 0.00237 | 0.00318 |
| CD4+CD45RA-CCR7-CD38+HLADR- | Activation | 0.002273 | 0.07478 | 0.00025 | 0.16532 |
| CD4+CD45RA-CCR7-CD38-HLADR- | Activation | 0.009447 | 0.04191 | 0.00211 | 0.42606 |
| CD4+ CD45RA+CCR7-CD95-CD28+ | Apoptosis | 0.000011 | 0.00002 | 0.00004 | 0.57365 |
| CD4+CD45RA-CCR7-CD95-CD28+ | Apoptosis | 0.000005 | 0.00029 | 0.00000 | 0.18062 |
| CD4+CD28- CD57+ | Senescence | 0.000059 | 0.00004 | 0.00054 | 0.29473 |
| CD4+CD28+ CD57+ | Senescence | 0.001620 | 0.00096 | 0.00749 | 0.21065 |
| CD4+CD45RA+CCR7-CD28+CD57+ | Senescence | 0.000233 | 0.00042 | 0.00028 | 0.83856 |
| CD4+CD45RA+CCR7-CD28-CD57+ | Senescence | 0.000354 | 0.00009 | 0.00679 | 0.21791 |
| CD4+CD45RA-CCR7+CD28-CD57+ | Senescence | 0.000070 | 0.00012 | 0.00014 | 0.67648 |
| CD4+CD45RA-CCR7-CD28-CD57+ | Senescence | 0.000015 | 0.00007 | 0.00002 | 0.54751 |
| CD4+CD45RA-CCR7-CD28+CD57+ | Senescence | 0.007908 | 0.00589 | 0.00777 | 0.69077 |
| CD4+CD45RA-CCR7-CD28+CD57- | Senescence | 0.008127 | 0.01805 | 0.00400 | 0.37209 |
| CD4+CD31+ | RTEs | 0.004656 | 0.00415 | 0.00430 | 0.76360 |
| CD4+CD45RA+CCR7+ CD31+ | RTEs | 0.000090 | 0.00035 | 0.00014 | 0.24828 |
| **CD8 T-cell subsets** | **Biological process** | **p-value (all groups)** | **p-value  (SNC vs. HIV)** | **p-value (SNC vs. pre-DAAs HIV/HCV)** | **p-value ( HIV vs. pre-DAAs HIV/HCV)** |
| CD8+ PD1+ Tim3+ | Exhaustion | 0.000017 | 0.00015 | 0.00004 | 0.09885 |
| **CD8+CD45RA+CCR7-PD1+Tim3+** | **Exhaustion** | 0.000028 | 0.00188 | 0.00009 | **0.00585** |
| CD8+CD45RA+CCR7+PD1-Tim3- | Exhaustion | 0.002608 | 0.00092 | 0.00752 | 0.72683 |
| CD8+CD45RA-CCR7+PD1-Tim3- | Exhaustion | 0.000150 | 0.00010 | 0.00059 | 0.74150 |
| CD8+CD45RA-CCR7+PD1+Tim3+ | Exhaustion | 0.000208 | 0.00012 | 0.00092 | 0.60711 |
| CD8+CD45RA-CCR7+PD1+Tim3- | Exhaustion | 0.000338 | 0.00022 | 0.00100 | 0.77844 |
| CD8+CD45RA-CCR7-PD1+Tim3+ | Exhaustion | 0.000131 | 0.00104 | 0.00008 | 0.32709 |
| CD8+ CD38+ HLADR- | Activation | 0.000224 | 0.03364 | 0.00007 | 0.02630 |
| CD8+ CD38- HLADR- | Activation | 0.000807 | 0.06095 | 0.00025 | 0.03608 |
| **CD8+CD45RA+CCR7-CD38+HLADR-** | **Activation** | 0.000005 | 0.16320 | 0.00002 | **0.00007** |
| **CD8+CD45RA+CCR7-CD38-HLADR-** | **Activation** | 0.000634 | 0.95438 | 0.00081 | **0.00135** |
| **CD8+CD45RA-CCR7-CD38+HLADR-** | **Activation** | 0.001527 | 0.36685 | 0.00100 | **0.00640** |
| CD8+CD45RA-CCR7-CD95-CD28+ | Apoptosis | 0.008795 | 0.00861 | 0.00591 | 0.92272 |
| CD8+CD45RA-CCR7-CD28+CD57- | Senescence | 0.002409 | 0.00656 | 0.00118 | 0.47879 |
